# Functionalisation of Polyvinylpyrrolidone on Gold Nanoparticles Enhances Its Anti-Amyloidogenic Propensity towards Hen Egg White Lysozyme

**DOI:** 10.3390/biomedicines5020019

**Published:** 2017-05-03

**Authors:** Tulika Das, Vidyalatha Kolli, Srijeeb Karmakar, Nandini Sarkar

**Affiliations:** Department of Biotechnology and Medical Engineering, National Institute of Technology Rourkela, Odisha 769008, India; dastulika05@gmail.com (T.D.); vidyalatha.kolli@gmail.com (V.K.);srijibkarmakar@gmail.com (S.K.)

**Keywords:** amyloid, thioflavint, gold nanoparticle, circular dichroism, lysozyme, disaggregation

## Abstract

Protein amyloids are characterized by aggregates that usually consist of fibres containing misfolded proteins and having a cross β-sheet conformation. These aggregates can eventually lead to several degenerative diseases like Alzheimer’s disease, amyotrophic lateral sclerosis (ALS), Huntington’s disease and Parkinson’s disease. The present study describes the effect of chemically synthesized polyvinylpyrrolidone (PVP)-conjugated gold nanoparticles (PVP-AuNps) on hen egg white lysozyme (HEWL) amyloids. The synthesized nanoparticles have been characterized using various biophysical techniques like Ultraviolet-Visible (UV-Vis) Spectroscopy, Transmission electron microscopy (TEM), X-ray diffraction (XRD) analysis, dynamic light scattering (DLS), zeta-potential measurement and Fourier transform infrared spectroscopy (FTIR). The aggregation studies showed that PVP acts as a partial inhibitor of HEWL amyloidogenesis. However, when conjugated to gold nanoparticle surface, it leads to complete inhibition of amyloid formation. Apart from inhibition, PVP-conjugated gold nanoparticles also exhibited a significant disaggregation effect on mature amyloids and hence can be exploited as an effective therapeutic agent against hereditary systemic amyloidosis.

## 1. Introduction

Amyloid fibrils are elongated protein aggregates with a rigid core. They are characterized by their long and straight morphologies, cross-β sheet structure and specific dye binding capacities [1]. Such aggregates of protein are related to several “misfolding disorders” where the soluble states of the protein are converted to highly organised fibrillar aggregates [2]. Such aggregates can be extremely detrimental to the cells since they can disrupt the vital cellular functions, overwhelm the protein degradation pathways and can also disrupt intracellular transport [3]. Also, the inclusion bodies within the cell can pose a major problem [3]. Amyloid fibril formation is mostly accompanied by a structural re-arrangement of native state into a β-sheet rich fibrillar conformation [4]. Amyloid fibrils are almost 10nm in diameter and are composed of 2–6 protofilaments. The cross-β motif characteristic of amyloid fibrils of all proteins consist of β-strand oriented perpendicular to and β-sheets parallel to the fibril axis [5,6]. It has been recently reported that proteins can be induced in vitro to form amyloids very similar in structure and toxicity as compared to disease-related proteins [7]. Amyloid fibril formation has an initial lag phase which is also called the nucleation phase. The lag phase is followed by a rapid elongation phase where the monomers or the oligomers form the protofibrils. Lastly, there is a stationary phase which occurs due to the formation of mature fibrils [8].

Nanoparticles are materials within a size range 1–100 nm which are composed of a core material and are often conjugated to a surface-modifying agent [9]. Inhibition of the fibrillation process by nanoparticles could be done either by increasing the lag phase (at the nucleation phase) or by decreasing the elongation phase (polymerization phase) [10]. In addition, nanoparticles can also slow down the rate of fibrillation by reducing monomers by diverting them from the polymerization pathway [10]. Experimental studies have shown that nanoparticles (cationic polystyrene nanoparticles) can stop the fibrillation process at the lag phase when added at the start of the experiment [10]. This indicates that after the critical nuclei is formed, the process of elongation is insensitive to the nanoparticles. Also, in the presence of nanoparticles, there is more peptide-nanoparticle interaction, which might result in the adsorption of the monomers or initially formed oligomers, thereby resulting in the inhibition of the fibrillation process [10]. One proposed hypothesis is that, owing to its high surface area to volume ratio, nanoparticles can divert the monomers from the polymerization pathway due to more of peptide-nanoparticle interaction, resulting in less availability of monomers for aggregation. Gold nanoparticles (AuNPs) in nanometer sizes in the range 1–100 nm exhibit unique optical, electronic and molecular recognition. They can be synthesized and characterized relatively easily. Also, it is an attractive contrast agent as it can be visualized in phase contrast and dark field microscopy [11]. Polyvinylpyrrolidone (PVP) is a completely nontoxic polymer and has a long polyvinyl backbone [12]. It serves as an excellent capping agent especially for noble metal particles. It is soluble in water as well as physiological solutions [13]. In the refolding study of bovine carbonic anhydrase B, PVP binds to the early molten globule-like refolding intermediate, thereby protecting the exposed hydrophobic groups [14]. This results in an increase in the refolding rate of bovine carbonic anhydrase. This is due to the high hydrophilicity of the amide groups present in the monomers of PVP [14]. Hence, for the present study we have selected PVP-capped gold nanoparticles for inhibition studies on HEWL amyloids. Point mutations in the gene coding for human lysozyme can give rise to huge amyloid deposits in the kidneys and liver [15]. Due to high-level structural and sequence similarity with human lysozyme, studies have been conducted in hen egg white lysozyme (HEWL) to study the fibrillation process [15]. According to our hypothesis, PVP, due to its high hydrophilicity, might inhibit the amyloid formation. Thus, when conjugated to the surface of gold nanoparticles it can result in complete inhibition of amyloid formation. Hence, PVP-AuNps may have therapeutic benefits against fibrillation.

## 2. Experimental Section

### 2.1. Materials

Gold (III) chloride hydrate, Sodium borohydride, Hen egg white lysozyme and 1-anilinonaphthalene-8-sulfonate (ANS) were purchased from Sigma-Aldrich (Kolkata, West Bengal, India). Polyvinyl pyrrolidone K-30 and Thioflavin-T were purchased from Himedia (Mumbai, Maharashtra, India). All other chemicals were of analytical grade.

### 2.2. Synthesis of PVP-Conjugated Gold Nanoparticles (PVP-AuNps)

Water-soluble gold nanoparticles were chemically synthesized using gold (III) chloride hydrate as the substrate and sodium borohydride as the reducing agent [13]. Polyvinyl pyrrolidone is used as the capping or stabilizing agent. The increase in size of the nanoparticles could be visually characterised by observing the change in the colour from yellow to light pink to brilliant red.

Sodium borohydride, being a strong reducing agent, can convert all the gold ions to neutral gold atoms as evident by the immediate colour change from light yellow to orange. A total of 588 µM of gold (III) chloride hydrate was mixed with 1mM of PVP in 20 mL of Milli-Q water and magnetically stirred at 600 rpm to obtain a clear homogenous PVP-Au^3+^ solution. The Au^3+^ ions in the composite were reduced by using sodium borohydride solution (1 mM, 600 µL). The solution was stirred for 40 min to obtain a stable gold nanoparticle solution. The synthesized PVP-AuNps were passed through a 0.4-micron filter for removal of any aggregates or dust particles. As a control study, bare gold nanoparticles (bAuNps) were also synthesized using the well reported Turkevich method [16,17]. For this, 200 µL of 1% gold chloride was added to 50 mL boiling distilled water kept at constant stirring. It was followed by the addition of 1 mL 0.05 M tri-sodium citrate. After 15 to 20 min, a deep ruby red colour appeared, confirming the synthesis of gold nanoparticles.

### 2.3. Characterization of PVP-Conjugated Gold Nanoparticles (PVP-AuNps)

#### 2.3.1. Ultraviolet-Visible (UV-Vis) Spectroscopy

Characterization of PVP-AuNps was done in a Perkin Elmer UV-Vis Spectrophotometer, Lamda-19. The scanning was done in the visible region 400–700 nm with scan speed of 350 nm/min. The PVP-AuNps (dispersed in water solvent) were directly scanned after synthesis.

Baseline correction was done using blank reference. The instrument was equipped with “UVWinlab” software for recording and analysis of the data.

#### 2.3.2. Size Distribution Analysis by Dynamic Light Scattering and Zeta-Potential Measurement

The hydrodynamic diameter of synthesized nanoparticles was measured using Zeta-sizernano-ZS (Malvern instrument, Malvern, UK). Data analysis was performed in automatic mode. The zeta potential of the nanoparticle was measured on a Zeta sizernano-ZS (Malvern instruments, Malvern, UK). All the glasswares used for the synthesis of nanoparticles were thoroughly rinsed with chromic acid. Milli Q water was used for the synthesis of PVP-AuNps to minimize any particulate contamination that may affect the DLS measurements. Also the sample was filtered through a 0.4-micron filter before sending for analysis.

#### 2.3.3. X-ray Diffraction (XRD) Analysis

The PVP-AuNp solution obtained was air dried on a glass slide to obtain a film. Characterization was done using a Multipurpose X-ray diffraction system (Rigaku Japan/Ultima-IV).

#### 2.3.4. Attenuated Total Reflectance-Fourier Transform Infrared Spectroscopy (ATR-FTIR)

The spectral characteristics of the liquid gold nanoparticle sample were collected in Attenuated Total Reflectance (ATR) mode in a Fourier transform infrared (FTIR) spectrometer. All spectra were recorded in the wavenumber range of 4000–400 cm^−1^. Spectra were referenced to a water background spectrum.

#### 2.3.5. Transmission Electron Microscope (TEM)

Morphology, size and shape of the synthesized AuNps were characterized using transmission electron microscopy (TEM). Sample preparation was done using the drop-casting method where 10 µL of the PVP-AuNp suspension was placed on carbon coated TEM copper grids and was allowed to air dry for 10min. Excess solution was removed using a blotting paper. Also, X-ray energy dispersive spectrometry (EDS) was done in TEM microscope for detecting the chemical elements.

### 2.4. Synthesis of Hen Egg White Lysozyme (HEWL) Amyloids

HEWL amyloidosis was induced by incubating the protein in 10 mM glycine-HCl buffer (pH = 2.0) at 55 °C, 30 rpm at a concentration of 35 µM [18].

### 2.5. Amyloid Inhibition and Disaggregation Study

Inhibition study was performed by incubating HEWL with PVP-AuNps and PVP at the start of the experiment (100 µL of synthesized PVP-AuNps was added to 30 mL of incubated HEWL of 35 µM concentration). Aliquots were taken at 24, 48, 72 and 96 h and analysis of inhibition was done by performing various spectrofluorometric assays and spectroscopic techniques. Similar assays were performed by incubating the mature amyloid fibrils with PVP and PVP-AuNps overnight at 37 °C for testing their disaggregating ability. The amount of PVP tested corresponded to the amount of PVP present in the PVP-AuNp sample.

#### 2.5.1. Thioflavin T (ThT) Binding Assay

Extrinsic fluorescence of Thioflavin T (ThT) (a benzothiazole dye), is used to identify and quantify amyloid fibrils in vitro. This technique has been widely used to monitor fibrillation kinetics in real-time [19,20]. When the dye is added to samples containing β-sheet-rich deposits, such as the cross-β-sheet quaternary structure of amyloid fibrils, it strongly fluoresces with excitation and emission maxima at approximately 440 and 490 nm, respectively [21,22]. Free ThT in an aqueous environment, however, has mostly weak fluorescence, with blue-shifted excitation and emission maxima at 350 and 440 nm, respectively [21].

For ThT assay, HEWL was first exposed to amyloidogenic conditions (pH = 2.0, T = 55·°C and 30 rpm) as reported and aliquots were drawn at intervals from the samples for estimation of amyloid formation through ThT fluorescence. HEWL amyloidosis was induced by incubating it in 10 mM glycine-HCl buffer (pH = 2.0) at 55 °C, 30 rpm and 35 µM concentration was maintained. A stock of 2.5 mM ThT was prepared at 10 mM phosphate buffer (pH = 6.5). Samples for fluorometry were prepared by adding 10 µL ThT stock solution to appropriate volume of incubated HEWL to make final HEWL concentration and volume 10 µM and 1000 µL respectively in 10 mM phosphate buffer (pH = 6.5). Samples prepared in this way were incubated for 30min at room temperature before taking emission scan from 460—610 nm.The excitation wavelength was kept at 450 nm. ThT assay was performed for testing the effect of PVP and PVP-AuNps on HEWL amyloids individually. To test the quenching effect of gold nanoparticles on ThT fluorescence, mature fibrils were taken and ThT fluorescence was measured. To the same fibrils PVP was added in appropriate quantity and immediately ThT fluorescence of the sample was measured again and the two data were compared. Any reduction in the latter sample will indicate quenching effect of the gold nanoparticles.

#### 2.5.2. Circular Dichroism (CD) Spectroscopy

Circular Dichroism CD measurements were done on a Jasco J1500 Spectropolarimeter equipped with a constant temperature cell holder. Conformational changes in the secondary structure of the HEWL were monitored in the far UV region between 190—250 nm with a HEWL concentration of 5 μM in a cuvette of 1mm path length [23]. The secondary structure of the HEWL was predicted using integrated software named “secondary structure predictor”.

#### 2.5.3. Fluorescence Microscopy

Fluorescence images were taken using a fluorescence microscope (Olympus, Gurgaon, Haryana, India) under 10× magnification. Dye used for fluorescence detection is ThT. About 10 µL of 1 mM ThT dye was added to 5 µL of test sample placed on a glass slide and covered with cover slip. To prevent quenching, the samples were kept in dark.

#### 2.5.4. ANS Binding Assay

Change in the exposed hydrophobic surface of the amyloid fibrils on addition of PVP and PVP-AuNps was monitored by ANS binding study [24]. A stock solution of 2.5 mM ANS was freshly prepared by dissolving appropriate amount of ANS in 50 µL of methanol and diluting with 950 µL of distilled water. About 180 µL of this ANS stock was added to the incubated HEWL sample such that ANS was in 100-fold molar excess of HEWL. The mixture was diluted to 1mL using distilled water and incubated in dark for 30 min prior to fluorescence scan. Fluorescence measurements were taken using Perkin Elmer, LS 55 Spectrofluorometer with excitation at 380 nm and emission scan from 390–600 nm. Slit widths were set at 5 nm for both excitation and emission and the scan rate set at 1200 nm/min.

#### 2.5.5. Prediction of Amyloidogenic Regions of the HEWL and Prediction of Binding Site of Polyvinylpyrrolidone

FoldAmyloid (an online web server) was used to predict the amyloidogenic regions of HEWL. It does the prediction by analyzing the amino acid sequence of the polypeptide chain by considering the physicochemical properties of amino acids [25]. The crystal structure of hen-egg white lysozyme was downloaded from Protein Databank (PDB ID-3WUN). Crystallographic structure with high resolution (2.4 A) was used for molecular docking study. Protein was visualized and ligands (Na^+^ and Cl^−^) were removed using Chimera v1.10.2. AutoDocking was performed in AutoDock 4.2 and the docked complex visualized in Chimera v1.10.2. LIGPLOT analysis gives 2D representations of ligand-protein interactions [26].

#### 2.5.6. Transmission Electron Microscopy 

Transmission electron microscopy (TEM) is used to detect the presence of fibrils using phosphotungstic acid as the staining dye [27]. A solution of 2% phosphotungstic acid (PTA) was prepared in Distilled De-Ionized (DDI) water. A total of 3 μLof HEWL sample was placed on the grid. Immediately 3 μL of staining solution (PTA) was placed on the grid. After 3 min, excess solution was wicked away and air dried. The grids are imaged using an electron microscope operating at 80 keV.

## 3. Results

### 3.1. Synthesis and Characterization of PVP-AuNps

PVP-capped gold nanoparticles were synthesized by mixing PVP (1 mM) in Milli-Q water(20 mL) with gold(III) chloride hydrate (588 µM) and magnetically stirred to obtain a clear homogenous solution of PVP-Au^3+^. Au^3+^ ions in the solution were then reduced using a strong reducing agent sodium borohydride (600 µL, 1 mM). The solution of PVP-AuNps obtained remained stable for a long duration (3–4 months) as checked by dynamic light scattering (DLS) measurements. Further, the characterization of the synthesized PVP-AuNps was done using various biophysical techniques like UV-Vis spectroscopy (Figure S1), Dynamic light scattering (DLS) (Figure S2, Table S1), zeta potential (Figure S3), X-ray diffraction (XRD) analysis( Figure S4, Table S2), Fourier transform infrared spectroscopy (FTIR) (Figure S5), and imaging by Transmission electron microscopy (TEM) (Figures S6 and S7). UV-Vis Spectroscopy of PVP-AuNps showed heavy absorbance at 539 nm of 1.4236 a.u, indicating formation of gold nanoparticles. Particle size analysis was done by dynamic light scattering which gave a peak maxima at 120.7 nm. The zeta potential value of −7.32 indicates that the nanoparticle is slightly negatively charged. XRD and FTIR analysis shows fingerprints of both gold and PVP, pointing towards the appropriate functionalisation of the gold nanoparticles with PVP. TEM images clearly show the morphology of the PVP-AuNps and EDS spectra gives us the elemental analysis.The details of characterization of the synthesized nanoparticles are shown in the supplementary data.

### 3.2. Thioflavin T Binding Assay

**Figure 1:**
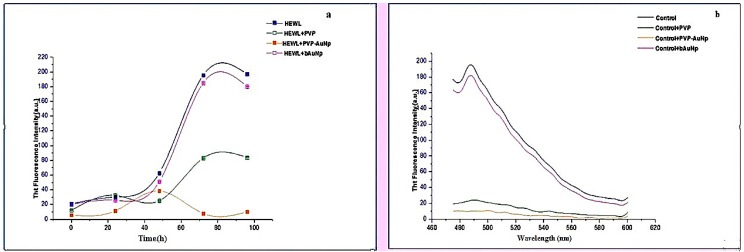
(**a**) Thioflavin T (ThT) binding assay: inhibition study; (**b**) ThT binding assay: disaggregation study—polyvinylpyrrolidone (PVP) and PVP-capped gold nanoparticles were added to mature hen egg white lysozyme (HEWL) fibrils and disaggregation readings were taken after 24 h. Au-NP: gold nanoparticle.

Thioflavin T has been used extensively for characterizing the presence of amyloid fibrils. It is a benzothiazole dye that gives enhanced fluorescence upon binding to amyloid fibrils. Figure 1a shows the aggregation kinetics of amyloid fibril formation by incubating HEWL with PVP as well as PVP-AuNps using time-dependent ThT fluorescence. PVP-incubated HEWL samples exhibited significant decrease in ThT fluorescence indicating a potential role of PVP in inhibiting HEWL amyloidogenesis. However, PVP-AuNp-incubated HEWL samples exhibited complete absence of ThT fluorescence throughout the incubation period, suggesting complete inhibition of amyloid formation. The study was continued until 96 h after mature amyloid fibrils were formed (as evident from the stationary phase). Bare gold nanoparticles (bAuNps) were used as control for the study and it was found to have no effect on amyloid inhibition of HEWL. A disaggregation study was also conducted by incubating mature HEWL amyloids with PVP and PVP-AuNps overnight at 37 °C and then, measuring ThT fluorescence, as shown in Figure 1b. Maximum disaggregation was observed by incubating 100 µL of synthesized PVP-AuNps to the sample of mature amyloids. Further, PVP alone appears to be quite effective in its ability to disaggregate the amyloids.

### 3.3. HEWL Secondary Structure Determination: Circular Dichroism Spectroscopy

**Figure 2:**
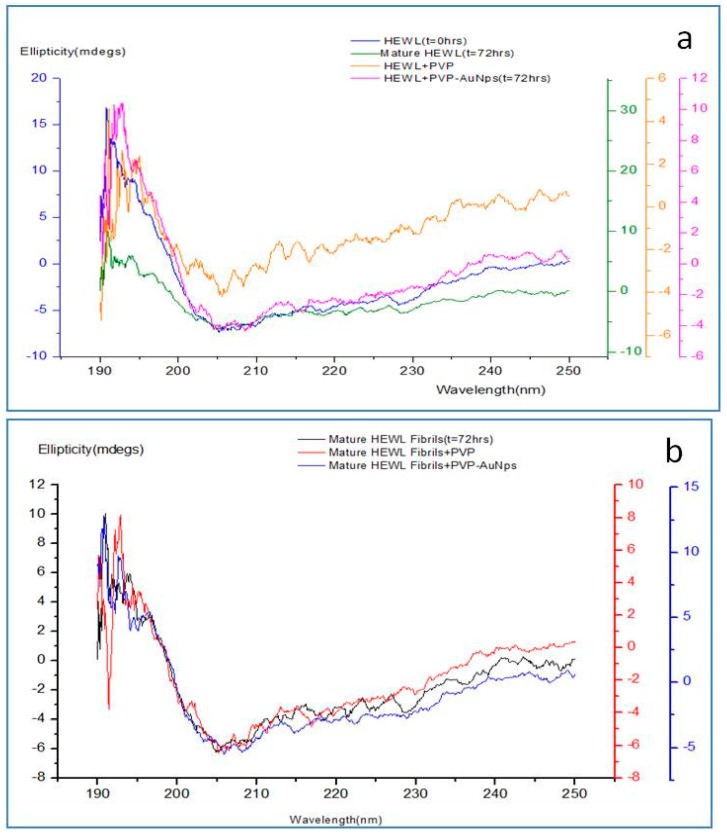
Far Ultraviolet-Circular Dichroism (UV-CD) spectra of (**a**) monomeric HEWL as well as HEWL incubated under amyloidogenic conditions in presence and absence of PVP and PVP-AuNps after 72 h of incubation (**b**) mature HEWL fibrils with and without addition of PVP and PVP-AuNps.

The chromophore that provides information about the peptide backbone in proteins is the peptide bond [28]. CD spectra is therefore recorded at the far-UV region (190–250 nm), the region where the peptide bond absorbs light [29]. Figure 2a shows the CD spectra of monomeric HEWL as well as HEWL incubated under amyloidogenic conditions in presence and absence of the nanoparticles. Similarly, Figure 2b shows CD spectra of mature fibrils and fibrils on addition of PVP and PVP-AuNps. HEWL incubated in glycine HCl to 50.8% and no significant change in α helix content, depicting the formation of amyloids. However, HEWL incubated with PVP under same conditions exhibited drastic decrease in α -helix content buffer (pH = 2.0) at a temperature of 55 °C for 72 h showed a significant increase in β sheet from 36.2%and slight decrease in β- sheet content (from 36.2% at 0 h to 40.9% at 72 h), as seen in Table 1 and Table 2. Also, HEWL at 72 h is more in the random coil conformation (49.6%) On the contrary, HEWL incubated with PVP-AuNps shows an increase in α helix content from 16% at 0 h to 28.1% at 72 h, and a significant decrease in β sheet content from 36.2% at 0 h to 10.1% at 72 h. Similar to the inhibition data, disaggregation effect by PVP-AuNps was also followed by drastic increase in α helix content of HEWL amyloids (from 16.0% in control to 36.4% in incubated sample) and a decrease in β sheet content (from 50.8% in control to 25.1% in incubated sample). The consistent decrease in beta sheet content of HEWL in presence of PVP-AuNps both during the inhibition and disaggregation studies further supports the anti-amyloidogenic propensity of PVP-AuNp since amyloidogenesis is accompanied by an increase in β sheet content in proteins [4].

### 3.4. Fluorescence Microscopy

**Figure 3:**
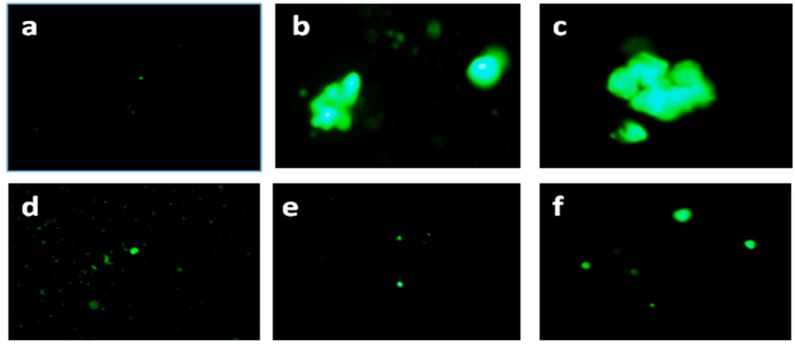
Fluorescence microscopic images (10×) of (**a**) monomeric HEWL; (**b**,**c**) Control:HEWL incubated under amyloidogenic conditions (*t* = 72 h); (**d**) HEWL+PVP incubated under amyloidogenic conditions (*t* = 72 h); (**e**,**f**) HEWL+PVP-AuNps incubated under amyloidogenic conditions (*t* = 72 h).

Thioflavin T (ThT) is used as dye to visualize the presence or fibrillation of misfolded protein aggregates or amyloids. The enhanced fluorescence upon binding to amyloid fibrils can be observed by fluorescence microscopy [30] (10× magnification), as shown in Figure 3. Under the same magnification, HEWL incubated with PVP as well as PVP-AuNps, exhibited significantly less ThT fluorescence compared to the control sample (HEWL fibrils) suggesting inhibition of amyloid formation. This study does not provide any quantitative measure of amount of amyloid formed or inhibited, however the comparative analysis clearly depicts the inhibitory effects of PVP as well as PVP-AuNps towards HEWL amyloidogenesis. Although, the ThT fluorescence emission of HEWL here in the presence of PVP and PVP-AuNps (Figure 3d–f) seems to be more or less same, the ThT assay data clearly depicts the enhanced inhibitory effect of PVP when functionalised on Au-Nps.

### 3.5. 1-Anilinonaphthalene-8-sulfonate (ANS) Binding Assay

**Figure 4:**
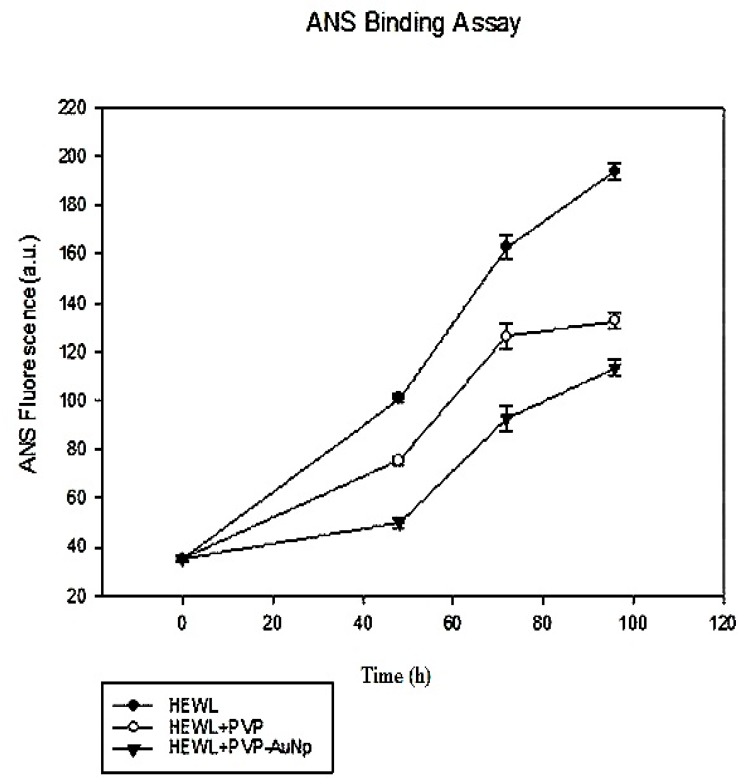
1-Anilinonaphthalene-8-sulfonate (ANS) fluorescence vs. time curve for HEWL (filled circle), HEWL incubated with PVP (hollow circle) and HEWL incubated with PVP-AuNps (filled inverted triangle).

ANS is a fluorescent dye that binds to the solvent exposed hydrophobic surfaces, such as those that is found in partially folded intermediate (molten globule state). When bound to such hydrophobic surfaces there is an 8–10 fold increase in the ANS fluorescence intensity. Also, ANS has weak affinity for native or completely unfolded protein state. Thus, ANS binding can be a useful probe for analysing the protein conformational state [31]. Figure 4 shows a time-dependent ANS binding assay of HEWL fibrils incubated with PVP as well as PVP-AuNps. It can be observed that in the control sample, a gradual increase in ANS fluorescence is found with time which is consistent with earlier reports indicating that aggregation is associated with increase in exposed hydrophobic patch. However, with PVP the increase in ANS fluorescence is not as great, and it is even less with PVP-AuNps. This indicates that PVP on binding with HEWL promotes alteration in conformation with less exposed hydrophobic patch. Further, in the presence of PVP-AuNp the decrease in ANS fluorescence is even greater, indicating that due to functionalization of PVP on the surface of AuNps, a greater number of PVP molecules are available to bind with HEWL and hence more HEWL molecules are altered in conformation, making them less prone to aggregation. PVP may act as chaperones and prevent aggregation by binding to the exposed hydrophobic patches in HEWL and thereby prevent further aggregation. To confirm that the reduction in ThT and ANS fluorescence was due to amyloid inhibition effect and not due to the quenching effect of AuNps, a small experiment was performed. Mature amyloid fibrils were formed, ThT data was taken and then appropriate volumes of bare gold nanoparticles (bAuNps) were added to the sample. Again, ThT data was taken immediately. No reduction in fluorescence emission was seen (data not shown) which confirms that AuNps do not have quenching effect in this fluorescence range. The decrease in ThT and ANS intensity observed in presence of PVP and PVP-AuNps may also arise due to competitive binding of these with HEWL, thereby preventing binding of the dyes. Hence, to confirm the anti-amyloidogenic effects of these compounds towards HEWL, further studies were done, such as TEM.

### 3.6. Computational Methods: Prediction of Amyloidogenic Regions of the HEWL and Prediction of Binding Site(s) of PVP

**Figure 5:**
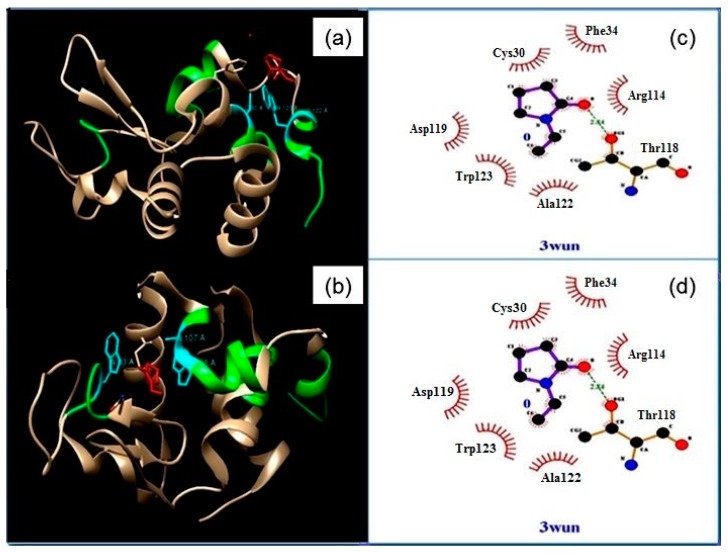
Docked Complex of HEWL and PVP showing the best two conformations (**a**,**b**) with binding energies −2.58 and −2.50 (green—amyloidogenic regions of HEWL, red—ligand, cyan—interacting residues); (**c**,**d**) Corresponding LIGPLOT analysis for determining the interacting residues of HEWL and the type of interaction.

FoldAmyloid is used to predict the amyloidogenic regions of the hen egg white lysozyme protein [27]. A region is predicted as being amyloidogenic if the average value of the parameter over that region is greater than threshold and the region is greater or equal in size to the frame. Four amyloidogenic regions were found in the protein chain—residues: 26–30, 60–64, 106–113, and 121–129. Figure 5 shows the best two docked conformations of PVP and HEWL with binding energies −2.58 and −2.50. LIGPLOT Analysis was done for determining the interacting residues of HEWL and the type of interaction. The ligand interacts with Cys 30 (C), Trp 123 (W), and Ala 122 (A) in conformation (a) and Ala 107 (W), Trp 108 (W), Trp63 (W) in conformation (b). The above-mentioned residues fall in the amyloidogenic region of HEWL as predicted by FoldAmyloid. Thus, the docking study further confirms that PVP has the ability to interact with the amyloidogenic regions of HEWL and thereby block aggregation. Although the structure of HEWL is not known in the pH value where it tends to form amyloid, we have docked PVP with the available structure of HEWL. Assuming that the vicinity of the amyloidogenic regions maintains compound-binding potential even after change in pH, our results reveal that PVP exerts its anti-amyloidogenic effect by binding to the aggregation-prone regions in HEWL through mostly hydrogen and hydrophobic interactions and thereby blocking aggregation.

### 3.7. Transmission Electron Microscopy (TEM)

**Figure 6:**
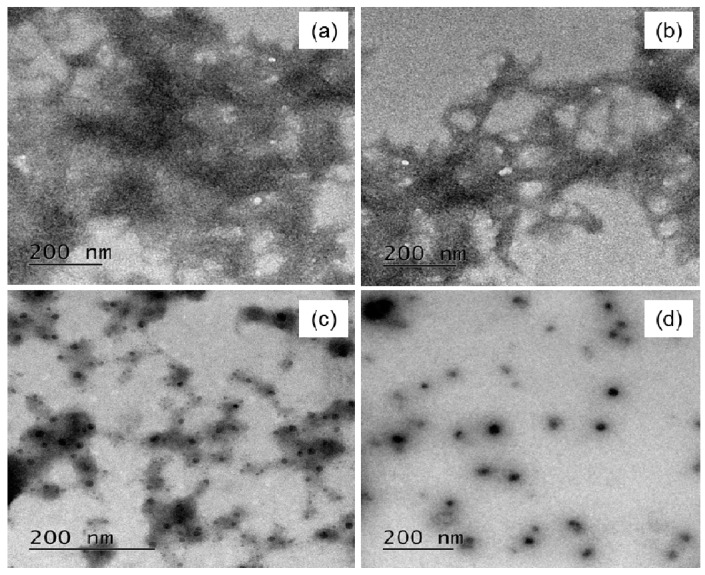
Transmission electron microscope (TEM) images of (**a**), (**b**) HEWL Amyloids (**c**) HEWL + PVP (**d**) HEWL + PVP-AuNps. Images were taken after 72 h of incubation.

Figure 6a,b shows the TEM images of mature HEWL amyloids, indicating dense fibrillar aggregates. Figure 6c shows the TEM images of HEWL incubated with PVP alone where some extent of inhibition is observed as evident from the small aggregates. Figure 6d shows the TEM images of HEWL incubated with PVP-AuNps where almost complete inhibition is observed. Thus, the TEM images confirm the inhibitory effects of PVP and PVP-AuNps towards HEWL amyloidogenesis. Further, PVP seem to have improved inhibitory effect when conjugated to Au-Nps, as seen from the TEM images.

## 4. Discussion

PVP-capped gold nanoparticles have been synthesized by wet chemical method and characterized using different biophysical techniques like DLS, XRD, UV-Vis Spectroscopy, FTIR and Field-Emission Scanning Electron Microscopy (FE-SEM). The synthesized nanoparticles have been tested upon HEWL amyloids: an inhibition and disaggregation study has been conducted by performing spectrofluorometric assays. PVP-AuNps lead to complete inhibition of HEWL amyloid formation compared to PVP alone, which leads to partial inhibition, as obtained from the ThT assay and fluorescence microscopy images. Another study by Dubey et al., also suggests that surface functionalisation of aromatic amino acids on gold nanoparticles increases their anti amyloidogenic potential towards insulin by making the binding site at the ligand molecules more accessible to the HEWL molecules [32]. Apart from inhibition, PVP-AuNps also exhibited disaggregation potential on mature HEWL amyloids. Inhibition and disaggregation of fibrils by PVP-AuNps were accompanied by similar type of conformation changes in HEWL, characterised by decrease in beta sheet content and increase in a helix content of HEWL. This suggests that the nanoparticles, on interaction with HEWL, are significantly altering their conformation, making them less prone to aggregation. However, PVP alone seems to induce different type of conformational changes in HEWL both during inhibition and as well disaggregation studies, which is characterized by a significant decrease in α helix content and an increase in random coiled structure. This suggests that when PVP is alone, it acts as a different ligand compared to when PVP is conjugated to AuNp, inducing different conformational alterations in HEWL and the latter seem to be more efficient in preventing as well as disaggregating amyloids. Results from ANS binding assay also indicate that the presence of PVP and PVP-AuNps alters HEWL into a lesser hydrophobic surface exposed conformation. Other studies have also reported inhibition of amyloid formation being accompanied by reduction in beta sheet content and ANS binding capacity of the protein [33,34]. However, the extent of decrease in ANS fluorescence was more in samples incubated with PVP-AuNps compared to samples incubated with only PVP. This can be explained by two possibilities: (1) due to local increase in PVP population as a result of conjugation on AuNp surface, more PVP molecules are accessible to HEWL for structural alteration resulting in decrease in ANS binding; and (2) PVP when conjugated to AuNps acts as a different ligand compared to PVP alone, inducing a different type of conformational change in HEWL. TEM images further confirm that PVP and PVP-AuNps act as inhibitors of HEWL amyloidogenesis, with the latter being more efficient. A Docking study was also performed to analyse the interaction of PVP with HEWL which showed that PVP interacts with the amyloidogenic region of HEWL as predicted by FoldAmyloid.

**Figure 7:**
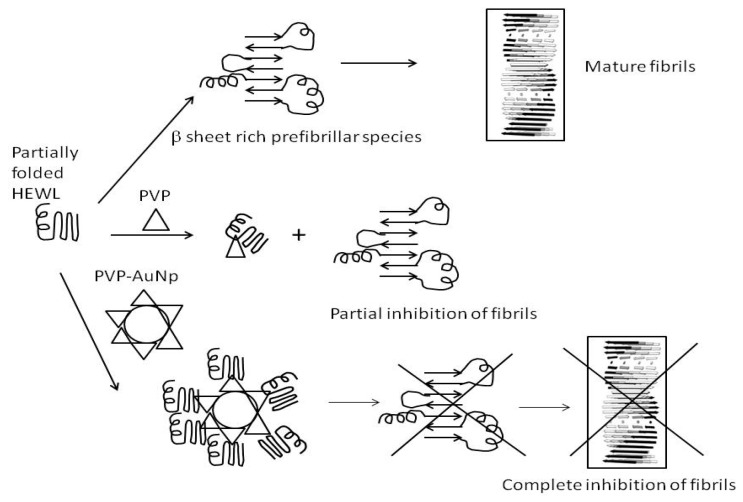
Schematic representation of the inhibitory effect of PVP and PVP-AuNps on HEWL amyloidogenesis.

Thus it can be concluded from the experimental and computational data that PVP acts as an inhibitor of amyloid formation. However, the inhibition is not complete since all the PVP molecules cannot access the aggregation-prone partially folded monomers. However, when the PVP molecules are conjugated to gold nanoparticles, the local concentration of the PVP molecules increases due to high surface area of the nanoparticle surface making it better accessible to the aggregation-prone intermediate. Further, it can be inferred, that the binding of PVP with the HEWL intermediates is followed by conformational changes in the HEWL molecules such as decrease in beta sheet, increase in alpha helix and reduced hydrophobic surface, making them less prone to aggregation. Moreover, PVP when conjugated to Au-Nps acts a different ligand compared to PVP alone, inducing different conformational changes in HEWL. A schematic representation of the role of PVP-AuNps as inhibitors of HEWL amyloidogenesis is shown in Figure 7. Thus these studies conclude that PVP-coated gold nanoparticles act as effective inhibitory and disaggregating agents against HEWL amyloids and thus can serve as potential therapeutics against lysozyme amyloidosis.

## Figures and Tables

**Table 1:** Monitoring changes in Hen Egg White Lysozyme (HEWL) secondary structure using Cicular Dichroism (CD): amyloid inhibition study.

Sample	Time (h)	Helix (%)	Beta (%)	Turn (%)	Random (%)
HEWL	0	16.8	36.2	3.4	43.7
	48	12.2	51.4	0.0	31.8
	72	16.0	50.8	0.0	33.2
HEWL+PVP	0	16.8	36.2	3.4	43.7
	48	8.3	40.6	3.2	47.9
	72	0.7	40.9	8.8	49.6
HEWL+PVP-AuNps	0	16.8	36.2	3.4	43.7
	48	19.7	31.2	7.0	42.1
	72	28.1	10.1	23.7	38.2

**Table 2:** Monitoring changes in HEWL secondary structure using CD: amyloid disaggregation study.

Sample	Control (Mature HEWL Amyloids)	PVP + Control (after 24 h)	PVP-AuNps + Control (after 24 h)
Helix	16.0	8.2	36.4
Beta	50.8	39.2	25.1
Turn	0.0	5.7	0.0
Random	33.2	46.9	38.5

## Supplementary Materials

The supplementary materials are available online at www.mdpi.com/2227-9059/5/2/19/s1.
