# Supplementary material for: Functionalisation of Polyvinylpyrrolidone on Gold Nanoparticles Enhances Its Anti-Amyloidogenic Propensity towards Hen Egg White Lysozyme
Source: Biomedicines. 2017 May 3;5(2):19. doi: 10.3390/biomedicines5020019 (PMC5489805; doi:10.3390/biomedicines5020019)
Supplement: Supplementary file 1 [file biomedicines-05-00019-s001.pdf]

Supplementary Data

1. Characterisation of the synthesized nanoparticles

1.1. **Ultraviolet-visible** *(UV-Vis) Spectroscopy*

Gold nanoparticles display a single absorption peak in the visible range between 510—550nm. This gives a brilliant wine red colouration which varies according to size. The absorption peak shifts to a longer wavelength (red shifting) with increase in the nanoparticle diameter. Thus, spectrometry is an important aspect regarding nanoparticle characterization. In the present study, the absorption of gold nanoparticle was measured in a double-beam spectrophotometer and the absorbance scan was done from 400—700nm. The colloidal gold synthesized in the experiment showed heavy absorbance at 539nm of 1.4236 a.u. (Figure S1).

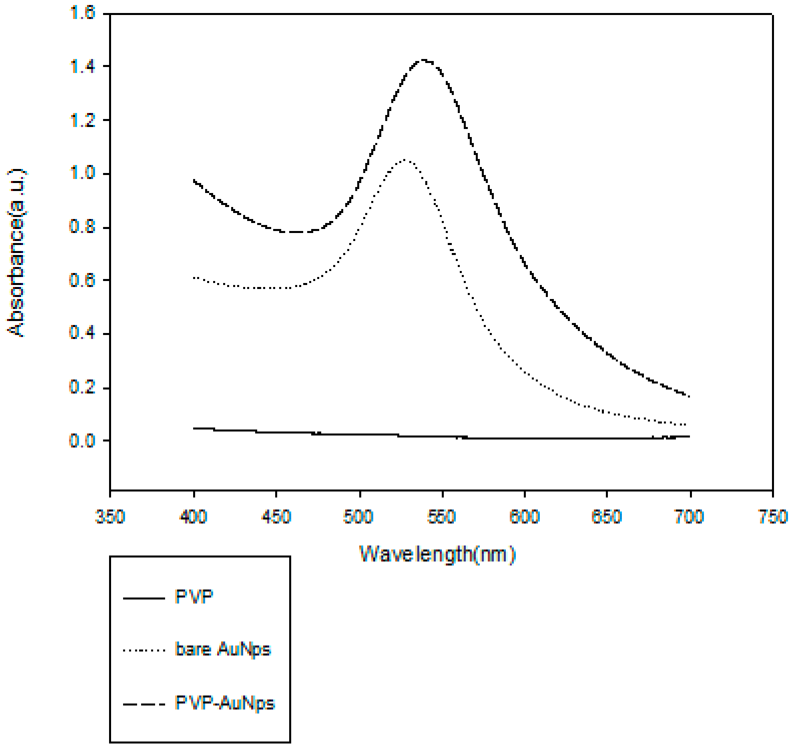

Figure S1. Absorbance spectra of Polyvinylpyrrolidone-Gold nanoparticle (PVP-AuNp).

1.2. Particle Size Analysis by Dynamic Light Scattering (DLS)

This measures the hydrodynamic size that can be defined as the size of a sphere that has the same translational diffusion co-efficient as that of the particle [1]. From the data (Figure 2) it is evident that for the majority of the nanoparticles, size ranges **from 50 to 150 nm with peak at 120.7 nm**. In addition there are certain particles of size less than 20nm. Thus, it can be concluded that the solution has particles of different sizes (both large and small sized nanoparticles). The Z-average value (d.nm)(average gold nanoparticle size) and the polydispersity index (PDI) value from the report are 95.04 nm and 0.406 respectively (Table 1). The PDI value is less than 0.7 thereby indicating that the

sample does not have a very broad size distribution and thereby ideal for dynamic light scattering (DLS) technique.

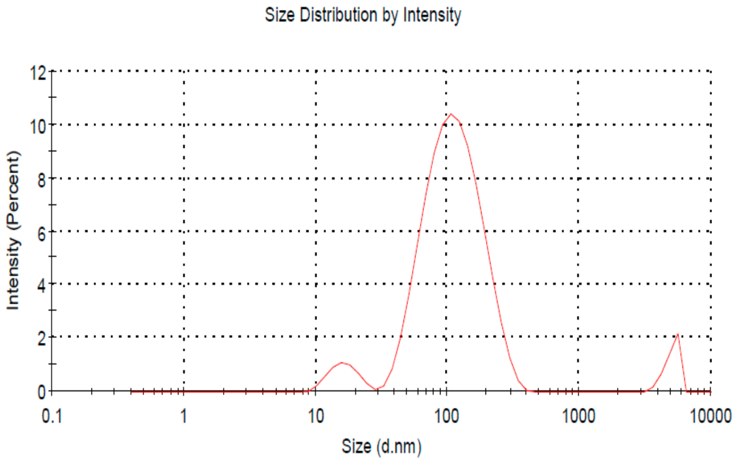

Figure S2. Size distribution graph of PVP-AuNp as obtained by DLS.

Table S1. Size Distribution results as obtained by DLS.

	Size(d.nm)	% Intensity	St Dev(d.nm)
Peak 1	120.7	90.6	58.13
Peak 2	16.61	4.9	4.031
Peak 3	5025	4.5	596.7

Z-Average (d.nm): 95.04, Pdl: 0.406

1.3. Zeta Potential Distribution Study

Electrostatic stabilization can be estimated by measuring the zeta potential, which is the charge at the surface of the electrical double layer [2].The zeta potential value from the report is –7.32 (figure 3a) which indicates that PVP-capped nanoparticles have low magnitude of zeta potential. A negative zeta potential value indicates that the particles are negatively charged. Thus, the nanoparticles coated with PVP remain dispersed in part by zeta potential and in part due to the steric hindrance created by the large PVP layer coating the surface of the particles. Cationic gold is electrostatically stabilized with the lone pair of electrons of oxygen in the carbonyl group and/or nitrogen in the pyrrolidone ring of PVP molecule.**The bare gold nanoparticles exhibited a zeta potential of –10.8 (figure 3b) which can be attributed to the adsorption of citrate ions on bAuNp surface.**

1.4. X-Ray Diffraction (XRD) Analysis

The XRD pattern shown in Figure 4 indicates fingerprints of both PVP and gold. The broad peaks at 2Θ=9-15° and 2Θ=19-25° are associated with the amorphous nature of PVP [3]. Diffraction peaks corresponding to (111), (200), (220) and (311) planes (Table 2) correspond to the face-centered-cubic (FCC) structure of gold. This depicts that the product is composed of pure crystalline gold and PVP which may be existing together while interacting with each other via covalent bonds. The crystal size can also be calculated from the XRD data using the Scherrer equation (1) [4], where Dp is average crystallite size, β is line broadening in radians, θ is Bragg angle, and λ is X-ray wavelength.

$$D_p = \frac{0.94\lambda}{\beta_{\frac{1}{2}} \cos\theta} \tag{1}$$

Considering the wavelength (ʎ) = 1.54056 and the peak corresponding to 2Θ=38.56, full width at half maxima (β) can be calculated. The β value can be calculated from the graph (=0.2 degrees). Thus, the average crystal size can be calculated from the above equation which comes to a value of 43.97nm.

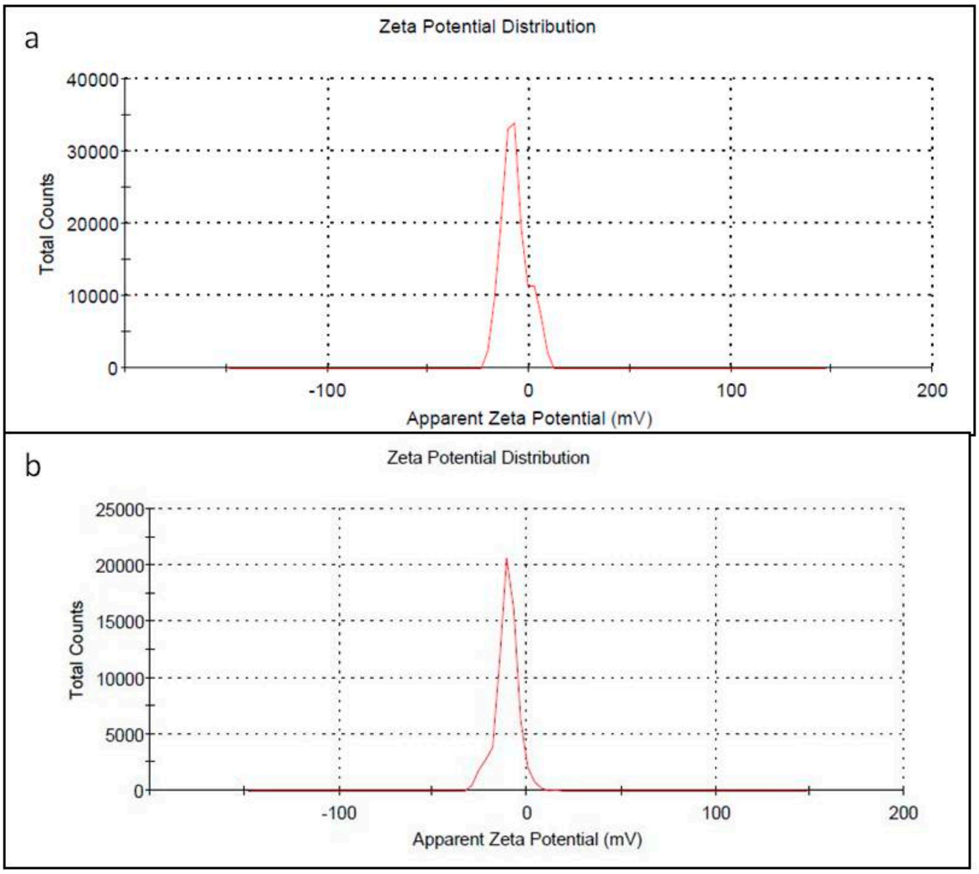

Figure S3. Zeta potential of (a) PVP-AuNp and (b) bAuNp

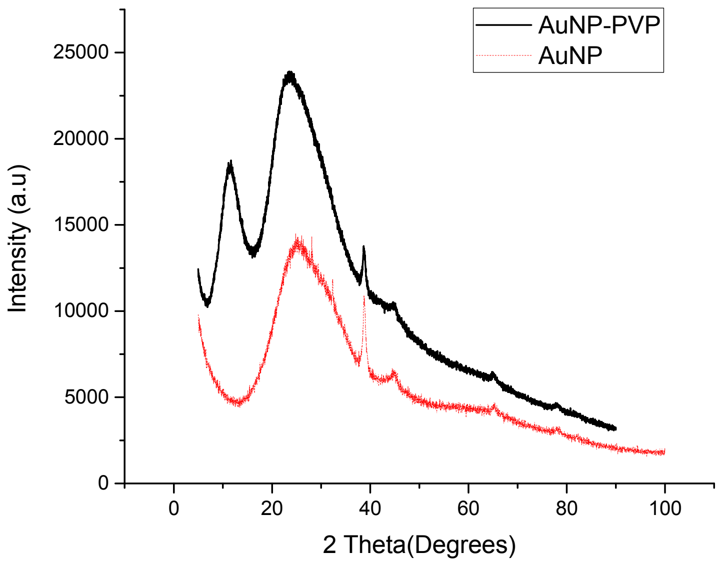

Figure S4. XRD scan of PVP-AuNp and bare AuNp.

Table S2. Analysis of Miller Indices as obtained by XRD.

2θ	θ (°)	θ (rad)	sinθ	sin²θ	ratio	K factor	h²+l²+k²	(hkl)
38.56	19.28	0.34	0.33	0.11	1.00	3.00	3.00	111
44.58	22.29	0.39	0.38	0.14	1.32	3.96	4.00	200
64.86	32.43	0.57	0.54	0.29	2.64	7.91	8.00	220
77.80	38.90	0.68	0.63	0.39	3.62	10.85	11.00	311

1.5. Fourier Transform Infrared (FTIR) Spectroscopy

The FTIR spectra of pure PVP and PVP-AuNps are shown in Figure 5. The absorption peaks at 1463cm^{-1} and 1424cm^{-1} were characteristic absorptions peaks of the pyrrolidinyl group of PVP. Also, peaks at 1643cm^{-1} and 1283cm^{-1}are due to the C=O and C–N vibrations in PVP [5]. The typical peaks in pure PVP also existed in PVP-AuNps indicating that PVP molecules were adsorbed onto the surface of gold nanoparticles. Thus, it can be concluded that appropriate functionalisation has taken place. The FTIR spectra of bare AuNps was also taken but no significant peaks were found in this range (data not shown).

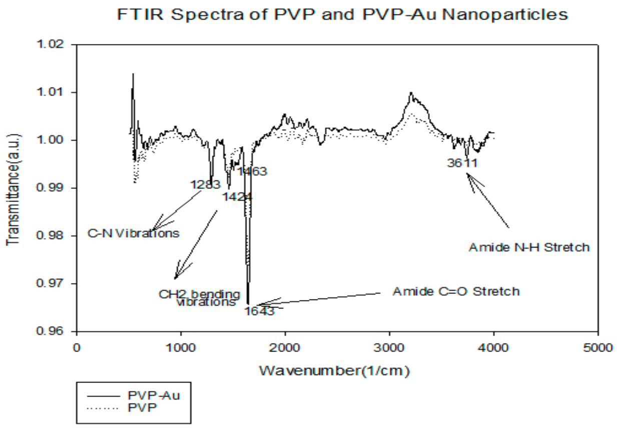

Figure S5. FTIR Spectra of PVP and PVP-AuNps.

1.6. Transmission Electron Microscopy (TEM)

The morphology of PVP-coated Au NPs can be studied with TEM. Figure 6 depicts microscopic images obtained from Au colloid with PVP molecules. Different sized nanoparticles (size ranging from 20 to 50nm) can be observed from the images. Size of PVP-AuNps is smaller in TEM images since it gives the core diameter unlike DLS which provides us with the hydrodynamic diameter of the nanoparticle. A high-resolution TEM image of PVP-coated gold nanoparticles displays the lattice fringes and thus confirms the crystalline nature of gold nanoparticles with interplanar spacing of 0.23nm.**TEM analysis of bAuNps exhibited nanoparticles of approx. 30 nm diameter. The relatively large size of PVP-AuNps compared to bAuNps can to be attributed to the surface coating with PVP which is a significantly large molecule.**

1.7. X-Ray Energy Dispersive Spectrometry (EDS)

Chemical analysis can be done in a TEM microscope by X-rays energy dispersive spectrometry (EDS). It can be used for the identification and quantification of chemical elements [6]. An EDS spectrum has peaks characteristic of chemical elements. EDS Spectrum is shown in Figure 7.

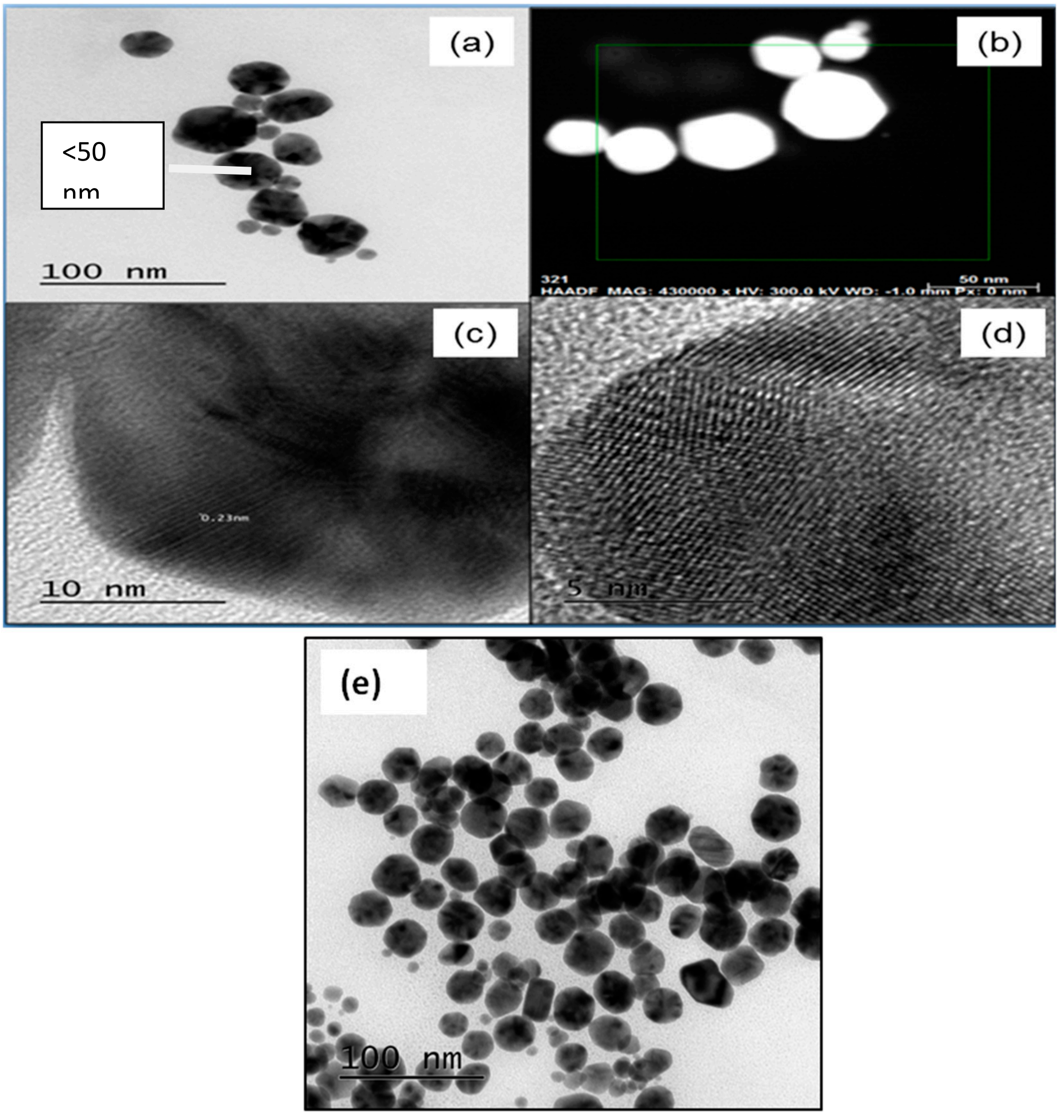

Figure S6. (a) TEM image (bright field) of PVP-AuNps. (**b**). TEM image (dark field) of PVP-AuNps (**c**), (**d**) high resolution TEM images displaying the lattice fringes of an XRD scan of PVP-AuNps. (**e**) TEM image (bright field) of bAuNps

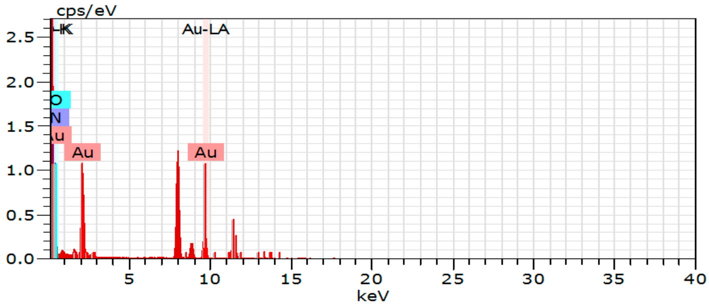

Figure S7. EDS Spectrum of PVP-AuNps.

References:

1. Pecora, R. Dynamic Light Scattering: Applications of Photon Correlation Spectroscopy, Plenum Press: Totowa, New York, **1985**.

2. Clogston, J.D.; Patri, A.K. Zeta Potential Measurement. In Characterization of Nanoparticles Intended for Drug Delivery McNeil, E.S., Eds.; Humana Press: Totowa, NJ, **2011**; pp. 63-70.
3. Ullah, M.H.; Hossain, T.; Ha, C. Kinetic studies on water soluble gold nanoparticles coordinated to poly (vinylpyrrolidone): isotropic to anisotropic transformation and morphology. J. Mater. Sci. **2011**, 46, 6988-6997.
4. Georgakilas, V.; Tzitzios, V.; Gournis, D.; Petridis, D. Attachment of magnetic nanoparticles on carbon nanotubes and their soluble derivatives. Chem. Mater.**2005**, 17, 1613-1617.
5. Wang, H.; Qiao, X.; Chen, J.; Wang, X.; Ding, S. Mechanisms of PVP in the preparation of silver nanoparticles. Mater. Chem. Phys. **2005**, 94, 449-453.
6. Wilson, A.R.; Lambrianidis, L.T. Compton-scattering X-ray artefacts observed in an STEM with high take-off angle EDS detector. J. Microsc. **1990**, 160, 1-7.
